# The Addition of *Lactobacillus* spp., Enrofloxacin or Doxycycline Negatively Affects the Viability of *Mycoplasma bovis* in Diluted Bovine Semen

**DOI:** 10.3390/ani10050837

**Published:** 2020-05-13

**Authors:** Ana García-Galán, Ángel Gómez-Martín, Esther Bataller, Jesús Gomis, Antonio Sánchez, Joaquín Gadea, Luis Alberto Vieira, Empar García-Roselló, Christian De la Fe

**Affiliations:** 1 Ruminant Health Research Group, Department of Animal Health, Faculty of Veterinary Sciences, Regional Campus of International Excellence “Campus Mare Nostrum”, University of Murcia, 30100 Murcia, Spain; ana.garcia25@um.es (A.G.-G.); asanlope@um.es (A.S.); cdelafe@um.es (C.D.l.F.); 2 Microbiological Agents Associated with Animal Reproduction (ProVaginBio) Research Group, Department of Animal Health and Public Health, Faculty of Veterinary Sciences, University CEU Cardenal Herrera of Valencia, CEU Universities, 46113 Valencia, Spain; esther.bataller@uchceu.es (E.B.); jesus.gomis1@uchceu.es (J.G.); empar@uchceu.es (E.G.-R.); 3 Physiology of Reproduction Research Group, Department of Physiology, Faculty of Veterinary Sciences, Regional Campus of International Excellence “Campus Mare Nostrum”, University of Murcia, 30100 Murcia, Spain; jgadea@um.es (J.G.); luisalberto.vieira@um.es (L.A.V.); 4 Institute for Biomedical Research of Murcia IMIB-Arrixaca, 30100 Murcia, Spain

**Keywords:** In vitro, viability, *Mycoplasma bovis*, diluted bull semen, enrofloxacin, doxycycline, *Lactobacillus* spp.

## Abstract

**Simple Summary:**

*Mycoplasma bovis* is an important infectious agent in cattle. The pathogen may cause a wide range of clinical signs, including mastitis, arthritis, pneumonia and reproductive disorders. Artificial insemination with contaminated semen may be a source of infection in infection-free areas or herds. Hence, the antimicrobials used in the preparation of seminal doses should be re-evaluated, or alternative measures to antimicrobials should be tested. This in vitro study aims to evaluate novel strategies to reduce the risk of the transmission of the pathogen through contaminated semen during artificial insemination. Hence, we assess the effect of the addition of (1) the antimicrobial enrofloxacin, (2) the antimicrobial doxycycline, or (3) a probiotic of human origin that contains acid lactic bacteria of the genus *Lactobacillus*, on the viability of *Mycoplasma bovis* in diluted bull semen in a Tris-citrate-fructose medium. The data show that the pathogen is negatively affected by the addition of 0.125 μg/mL of enrofloxacin, 0.0625 μg/mL of doxycycline, or the probiotic at a concentration of 3.24 × 10^6^ or 3.24 × 10^8^ colony-forming units/mL in diluted semen. Our results are promising in the field, as they may support new strategies to reduce the risk of the transmission of *Mycoplasma bovis* through artificial insemination.

**Abstract:**

*Mycoplasma bovis* is an important etiologic agent of bovine mycoplasmosis in cattle. Different transmission routes have been described, including those related to reproduction. The presence of mycoplasma in semen has led to its appearance in infection-free areas through artificial insemination (AI). Semen was recently reported to be the initial source of two *M. bovis* mastitis outbreaks in two closed dairy herds in Finland. This questions the effectiveness of the antimicrobials currently used in semen extenders to control the pathogens in contaminated semen. They should be re-evaluated, or alternative measures to antimicrobials should be tested to obtain *M. bovis-*free semen. This in vitro study aimed to assess different strategies to reduce the risk of transmission of *M. bovis* through AI technologies. The viability of *M. bovis* (PG45, NCTC 10131) in bull semen diluted (DS) in a Tris-citrate-fructose solution was tested, after the addition of enrofloxacin, doxycycline or a *Lactobacillus* spp.-based probiotic. The data show the susceptibility of the pathogen to the addition of 0.125 μg/mL of enrofloxacin or 0.0625 μg/mL of doxycycline and to the addition of the probiotic at a concentration of 3.24 × 10^6^ colony forming units (CFU)/mL or 3.24 × 10^8^ CFU/mL in DS. The Tris-citrate-fructose medium negatively affected the viability of *M. bovis*, although this effect was lower than that observed after the addition of the probiotic and antimicrobials (*p <* 0.05). Our results may support new strategies for reducing the risk of *M. bovis* transmission through AI.

## 1. Introduction

*Mycoplasma bovis* is an important infectious agent, which is distributed worldwide and is responsible for substantial economic losses in the cattle industry [1]. The pathogen may cause mastitis, pneumonia, arthritis, otitis media, keratoconjunctivitis, and reproductive disorders [2]. In bulls, *M. bovis* may cause orchitis, vesiculitis, and decreased sperm quality [3,4]. 

Different transmission routes have been described, including those related to reproduction. Even if bulls with clinical symptoms of *M. bovis* infection are not used as semen donors, the existence of asymptomatic carriers may lead to the spread of the bacterium inside infection-free areas through contaminated semen used for artificial insemination (AI). This may have recently occurred in Finland, where semen was reported to be the initial source of *M. bovis* mastitis outbreaks in two close dairy herds [5]. *Mycoplasma bovis* can be isolated from commercial frozen semen [6,7], and several experimental studies have shown that *M. bovis* can survive the in vitro fertilization processes, infect embryos, and interfere with fertilization by reducing the sperm penetration capacity [8,9]. 

Currently, semen extenders, employed to prepare bovine seminal doses, are supplemented with several antimicrobials to prevent the growth of semen contaminating bacteria, mainly acquired during the collection process. Penicillin, gentamicin, streptomycin, tylosin, spectinomycin, and lincomycin are among the most common antimicrobials added to semen extenders [10,11,12]. With regard to mycoplasmas, the addition of the same combination of gentamicin, tylosin, lincomycin, and spectinomycin has been effective or ineffective in different studies [13,14]. On the other hand, antimicrobials, such as enrofloxacin or doxycycline, have been shown to be efficient in inhibiting the growth of *M. bovis* in several in vitro studies [15,16]. However, the efficacy of these antimicrobials in bovine semen has not been assessed yet. Other tetracyclines, such as chlortetracycline and oxytetracycline, may be spermicidal for bovine sperm [17], but this has not been demonstrated for doxycycline. Furthermore, it has been shown in human patients that oral treatment with doxycycline for 10 days has no negative effect on sperm quality, compared to pre-treatment values. In fact, there may be a small benefit for patients with subnormal counts and motility [18].

Lacking a cell wall, mycoplasmas are very sensitive to environmental changes and especially to pH variations, which could be of interest in relation to the control of mycoplasma genital infections [19]. Indeed, the sensitivity of *Mycoplasma agalactiae* and *Mycoplasma mycoides* subsp. *capri* to the pH decreases in goat ejaculates and diluted semen was recently reported [20]. On the other hand, previous studies have demonstrated that lactic acid bacteria (LAB) of the genus, *Lactobacillus* spp., can inhibit the growth of human pathogenic bacteria through competition for resources, stimulation of the host immune system, and the production of hydrogen peroxide and acetic and lactic acids, which decrease pH [21,22]. LAB have been isolated in bovine semen [23], although their effect on the viability of bovine mycoplasmas is unknown. We believe that the viability of *M. bovis* in diluted bull semen (DS) may be affected by the addition of LAB as competing agents and pH acidifiers. 

Alternative control measures are needed to reduce the risk of the transmission of the pathogen through AI. Hence, the objective of this study is to assess the viability of *M. bovis* in experimentally contaminated DS, after the addition of (1) an antimicrobial (enrofloxacin or doxycycline), or (2) a *Lactobacillus* spp.-based probiotic at different concentrations. 

## 2. Materials and methods 

### 2.1. Semen Samples 

All animal procedures were performed following the EU Directive 2010/63/EU for animal experimentation and had the authorization of the Ethics Committee on Animal Testing of the University of Murcia (Number: 307/2017). 

Samples from nine Aberdeen Angus bulls, between two and four years of age, from a herd in Teruel (Spain), were collected for this study on three different days. Four samples were collected on day 1, three samples on day 2, and two samples on day 3. 

An ejaculate sample was obtained from each bull by electroejaculation [24]. In the first place, the animals were sedated with xylazine (3–4 mg/100 Kg Rompun® 2% i.m.; Bayer S.A., Barcelona, Spain). Then, the preputial area was washed and disinfected, and rectal ampoule emptying was carried out. An electroejaculator, with an automatic function (Electrojac 6, Humeco®) and a 63.5 mm three-electrode probe, was used. The electroejaculation program consisted of a consecutive series of increasing voltage, with two-second pulses, followed by a two-second pause. The ejaculates were then collected using a tempered glass collector in a graduated tube and diluted (1/1, 37 ºC) in a Tris-citrate-fructose medium (250 mM Tris, 88 mM citric acid, 14 mM fructose; 325–350 mOsm; pH 7.0) [25]. Then, the DS samples were transported in an airtight isothermal container to the laboratory in less than four hours.

The sperm motility of each DS sample was assessed using the computer-assisted sperm analysis system (ISAS, Proiser, Valencia, Spain) [26]. Samples with a motility below 50% or with contamination signs were discarded. Two samples on day 1, two samples on day 2, and two samples on day 3 were finally selected for the study. Each sample was adjusted to a concentration of 25 × 10^6^ cells/mL, and a pool of DS was prepared with the selected samples. 

### 2.2. M. bovis Inoculum 

The reference strain of *M. bovis* (PG45, NT 10131) was cultured in a modified broth SP4 medium [27], without any antimicrobial addition, supplemented with heat-inactivated horse serum (HIHS) instead of bovine serum, and incubated for 48 hours at 37 °C in a humidified atmosphere with 5% CO_2_. The initial concentration of the inoculum (1 × 10^9^ colony-forming units (CFU)/mL) was calculated as previously described [28]. 

### 2.3. Lactobacillus spp. Inoculums

A commercial lyophilisate of human origin, based on *Lactobacillus crispatus*, *Lactobacillus gasseri,* and *Lactobacillus brevis* (NS Femibiotic ®, Huarte, Pamplona, Navarra, Spain), was reconstituted in a modified broth SP4 and incubated for 24 hours at 37 °C. Two inoculums of *Lactobacillus* spp. (L1 and L2) were prepared at different concentrations (3.24 × 10^6^ and 3.24 × 10^8^ CFU/mL, respectively). 

### 2.4. Determination of Minimum Inibitory Concentration (MIC) 

The MIC of enrofloxacin (Fluka, Bio-Chemika, Missouri, USA) and doxycycline (Sigma-Aldrich, St. Louis, Missouri, USA) was calculated for the PG45 strain. A stock solution (1 mg/mL) of each antimicrobial was prepared by dissolving the powder in sterile distilled water, from which 12 serial double dilutions were made. To prepare enrofloxacin, 0.1 M HCL was added dropwise until dissolution occurred, and the correct final volume was obtained by adding sterile distilled water. A final range from 128 to 0.0625 μg/mL was tested. A stationary-phase culture of the PG45 strain was used for MIC assays. The mycoplasma culture was carried out in a PH medium [29], supplemented with sodium pyruvate (0.5%) and phenol red (0.005%), and the mycoplasma titers were determined using a previously described method [28]. MIC assays were carried out in 96-well microtiter plates using the microbroth dilution method [30]. Briefly, 25.6 µL of each antimicrobial dilution and 25 µL of the diluted *M. bovis* inoculum (10^3^–10^5^ CFU/mL) were added to 150 µL of the culture medium. After 48 h of incubation at 37 °C, the plates were examined for color change. MIC was defined as the lowest concentration of the antimicrobial capable of completely inhibiting the growth of the PG45 strain. This value was recorded for both antimicrobials and was 0.125 μg/mL and 0.0625 μg/mL for enrofloxacin and doxycycline, respectively. MIC assays were performed in duplicate. To accept the results, the MIC values had to be within one dilution, and the higher MIC value was used. 

### 2.5. Experimental Design 

Eleven in vitro experimental conditions (Table 1) were prepared, with a final volume of 1.5 mL, using DS and/or modified SP4 to investigate the *M. bovis* viability after exposing it to two antimicrobials, enrofloxacin or doxycycline, or different concentrations of *Lactobacillus* spp. Previously, the MIC of PG45 was determined for both antimicrobials. 

Each condition was prepared in a sterile eppendorf tube. DS was added to conditions 1, 2, 3, 4, 5, 8, and 9 to obtain the final volume of 1.5 mL. In conditions 6, 7, 10, and 11, a similar procedure was performed using SP4. 

Posteriorly, 40 μL of the *M. bovis* inoculum (1 × 10^9^ CFU/mL) was added to the conditions, followed by the addition of 40 μL of L1, 500 μL of L2 (3.24 × 10^6^ CFU/mL and 3.24 × 10^8^ CFU/mL, respectively: Table 1), or 24 μL of enrofloxacin or doxycycline (obtaining a final concentration of 0.125 μg/mL and 0.0625 μg/mL, respectively, with a final volume of 1.5 mL: Table 1). The eppendorf tubes were incubated in a thermoblock (VWR, Radnor) at 37 °C for 15 hours (15). After 15 minutes of incubation, 0 hour (h0) was established. The viability of *M. bovis* and LAB was determined at 0 and 15 h after exposure (h0 and h15, respectively). 

Besides, pH measurements were made using an electronic pH meter at both times (Hamilton Minitrode, Bonaduz, Switzerland). The pH values of the pools of DS and SP4 were considered as the initial pH (h0) of the conditions prepared for DS and SP4, respectively. The pH was measured in every condition separately at h15. 

The absence of mycoplasmas in the pools of the DS and SP4 medium was confirmed by the culture in SP4 agar plates. The plates were grown at 37 °C in 5% CO_2_ and examined daily under a light microscope. No colonies were observed after 15 days in incubation. *Mycoplasma bovis* was further discarded in the pool of DS by polymerase chain reaction (PCR) from DNA extracted from 200 μL of the culture [31,32]. 

Three replicates of this experiment were performed on different days. 

### 2.6. M. bovis Viability

Viable CFU/mL were determined at h0 and h15 in every condition contaminated with *M. bovis* (Table 1, *n* = 9), as previously described [28], but diluted in the modified SP4 broth. Counts were conducted on the SP4 agar, after incubation at 37 °C for 48 hours and in 5% CO_2_. For each replicate, counts were conducted in duplicate, with four dilutions for each contaminated condition, and at both times. Only counts with at least one visible mycoplasma were considered for statistical analysis.

### 2.7. LAB Viability

Viable LAB CFU/mL were determined in conditions 1 to 7 (Table 1) using a serial dilution method, as previously described [33]. In our study, dilutions were carried out in modified SP4 broth, and counts were conducted on MRS agar plates (MRS, Agar, Scharlau) after incubation at 37 °C for 24 hours. For each replicate, counts were conducted on one dilution for each condition and time. 

### 2.8. Statistical Analysis 

Counts of *M. bovis* and LAB were transformed as log (1 + C), where C was the count obtained (CFU/mL) for each analytical condition and organism. Statistical analysis was performed using a general linear procedure implemented in the program Statistical Analysis System Institute (SAS) [34], following the model:

Y_ijk_ = μ + S_i_ + C_j_ + T_k_ + CT_jk_ + e_ijk_ (1)

where Y_ijk_ = pH and log CFU/ml of *M. bovis* and LAB (dependent variables); μ *=* mean; S_i_ = sample effect; C_j_ = effect of analytical conditions; T_k_ = effect of time; CT_jk_ = effect of the interaction between the analytical condition and time; and e_ijk_ = residual effect.

## 3. Results 

In the proposed model, the condition itself, the time, and the interaction between the condition and the time had a significant effect on the pH and log CFU/mL of *M. bovis* and LAB. Table 2 shows the effect of each condition on *M. bovis* viability, regardless of the time, while Table 3 shows the effect of the time on the pH and the *M. bovis* and LAB viability in each condition separately. The addition of both concentrations of the probiotic and both antimicrobials to DS had a detrimental effect on the mycoplasma viability, since its concentration was lower (*p* < 0.05) than in untreated DS (Table 2, conditions 1, 4, 5, 8, and 9). However, the addition of the probiotic at a higher concentration (condition 5) had a greater impact (*p* < 0.05) on the pathogen viability than the addition of a less concentrated probiotic (condition 4) and was equivalent to the addition of enrofloxacin and doxycycline (conditions 8, 9). 

Considering the log CFU/ml of *M. bovis* and LAB variation over time (Table 3), a significant LAB growth was recorded in DS when adding L2 (condition 5), while a harmful effect on *M. bovis* viability was observed, since log CFU/mL decreased from 7.08 to 0.78 (*p <* 0.05). Besides, this effect on mycoplasma viability coincided with a decrease of the pH from 6.93 to 6.5 (*p <* 0.05). However, when adding the less concentrated probiotic, the pathogen survival was negatively affected, although no significant LAB growth was observed, and the pH remained constant (condition 4). 

Both L1 and L2 remained viable in DS (conditions 2 and 3), although there was no significant LAB growth, and the pH remained constant. On the other hand, neither L1 nor L2 grew significantly in the contaminated SP4 (conditions 6 and 7), while the pH decreased (*p <* 0.05) from 7.6 to 6.73 when L1 was added, and from 7.6 to 7.21 when L2 was added. Furthermore, the viability of *M. bovis* was not affected, and its concentration increased (*p <* 0.05) in the L2 condition. 

The viability of *M. bovis* was negatively affected (*p <* 0.05) over time in the untreated DS, since its concentration decreased from 7.53 to 5.89 (*p <* 0.05), while no significant variation was registered in the pH, which was 6.74 after 15 hours of incubation (Table 3, condition 4). However, this detrimental effect on the pathogen viability was lower (*p <* 0.05) than that observed after the addition of the probiotic and antimicrobials (Table 2, conditions 1, 4, 5, 8, and 9). Furthermore, the counts of LAB were made in this condition (Table 3, condition 1), even though it had not been supplemented with the probiotic.

Concerning the antimicrobial conditions, the viability of the pathogen was negatively affected (*p <* 0.05) in DS and SP4, while the pH remained constant in all of them (Table 3, conditions 8, 9, 10, and 11). 

The MIC value of PG45 for enrofloxacin was within the range of previously published studies applying microbroth dilution tests [30,35]. No published values are available for doxycycline. 

## 4. Discussion 

Our results show the in vitro susceptibility of *M. bovis* in DS after the addition of *Lactobacillus* spp. at a concentration of 3.24 × 10^6^ CFU/mL or 3.24 × 10^8^ CFU/mL and after the addition of 0.125 μg/mL enrofloxacin or 0.0625 μg/mL of doxycycline. While the Tris-citrate-fructose medium negatively affected the viability of *M. bovis*, this effect was lower than that observed after the addition of the probiotic and antimicrobials. 

Several studies have demonstrated the inhibitory role of *Lactobacillus* spp. against human pathogenic bacteria, and they are mainly used for the treatment of vaginal dysbiosis [21,22]. Lacking a cell wall, mycoplasmas are very sensitive to pH variations [19], and so we thought that the addition of *Lactobacillus* spp.-based probiotics could affect the viability of *M. bovis* in DS due to the pH decrease after LAB proliferation. Indeed, this phenomenon was observed after the addition of the probiotic at a concentration higher than 10^8^ CFU/mL. However, after adding the probiotic at a lower concentration, the viability of *M. bovis* was negatively affected, but no significant LAB growth nor pH decrease was observed. Nevertheless, the LAB counts were evidenced, so the competition for resources or the production of substances, such as hydrogen peroxide [21,22], could have affected the viability of the pathogen. 

Interestingly, the viability of *M. bovis* in untreated DS was seriously affected after 15 h in incubation. This is consistent with previous observations in goat DS contaminated with mycoplasmas [20]. These authors demonstrated that the acidic pH of goat ejaculate (pH ≥ 7) after dilution in the semen extender (pH ≤ 6) caused a detrimental effect on *M. agalactiae* and *M. mycoides* susbs *capri*. Furthermore, the glycolytic activity of sperm in the semen extender contributes to the acidification of the medium, which may negatively affect the viability of mycoplasmas present in DS [20,36]. On the other hand, we confirmed that *Lactobacillus* spp. strains remained stable and viable in DS, which could be due to the presence of nutrients in the semen extender or even synergies occurring with the saprophytic semen flora. In this sense, previous authors have described the presence of LAB in bovine semen [23]. This could explain why counts of LAB were made in the contaminated DS, without the probiotic addition. Indeed, the natural presence of LAB in bovine semen could lead to a detrimental effect on *M. bovis* viability. However, since the ejaculate is diluted for the preparation of the seminal dose, the possible effect of LAB on the pathogen viability could be reduced. The addition of LAB in DS could compensate for the effect of the dilution. 

The addition of the probiotic in SP4 did not negatively affect the viability of *M. bovis*. Indeed, LAB remained stable, and the concentration of *M. bovis* was significantly increased in condition 7, with L2. In this case, the significant decrease of pH in SP4 after the addition of *M. bovis* and the probiotic may be due to the metabolic activity of the viable bacteria present in the medium. 

On the other hand, the addition of the antimicrobials, enrofloxacin (0.125 μg/mL) or doxycycline (0.0625 μg/mL), may be an effective measure to reduce the presence of *M. bovis* in DS. Some authors have reported a reduced efficacy of fluoroquinolones and doxycycline under low pH conditions, so the acidic pH of DS (6.93) could have affected their antimicrobial activity [37,38]. Nevertheless, the conditions prepared in SP4 (pH 7.68) and DS (pH 6.93) were equivalent, and the viability of the pathogen was negatively affected in all of them (Table 2, conditions 8, 9, 10, and 11). 

To the authors’ knowledge, this is the first study that evaluates the effect of the addition of enrofloxacin, doxycycline, or the *Lactobacillus* spp.-based probiotic on *M. bovis* viability in bovine DS. Our results are promising in the field of bovine mycoplasmosis, as they may support new strategies for reducing the risk of *M. bovis* transmission through AI. 

Despite the promising results, this study has several limitations. (1) Only the reference strain PG45 was considered, since this is a preliminary study. Further analyses should consider the effect of the studied conditions on *M. bovis* field isolates. (2) The effects on the sperm quality (viability, motility, and morphology) of the addition of the antimicrobials and the probiotic were not evaluated. Further investigations should be conducted to examine the effects, if any, of those compounds on bull sperm quality. Those studies should consider not only the concentrations employed here, but also concentrations below and above, to obtain the lower effect on sperm quality and the higher effect on *M. bovis* viability. Nevertheless, the main objective of this study was to evaluate the effect of the addition of those compounds on *M. bovis* viability. Hence, the authors believe that the results reported in the present essay are significant and worthy of consideration. Further studies should be conducted to fill the aforementioned gaps. 

## 5. Conclusions 

In the present study, we report the in vitro susceptibility of *M. bovis* (PG45, NCTC 10131) to the addition of *Lactobacillus* spp. at a concentration of 3.24 × 10^6^ CFU/mL or 3.24 × 10^8^ CFU/mL and to the addition of 0.125 μg/mL enrofloxacin or 0.0625 μg/mL of doxycycline in DS. The semen extender, Tris-citrate-fructose, also negatively affected the viability of the pathogen, although to a lesser extent than the addition of the probiotic and antimicrobials. Our results are promising in relation to the control of bovine mycoplasmosis, as they may support new strategies for reducing the risk of *M. bovis* transmission through AI. 

## Figures and Tables

**Table 1:** Experimental conditions evaluated.

Condition	Description	*M. bovis*	LAB
1	DS _(1460 μL)_ + *M. bovis*_(40 μL)_	✓	✓ *
2	DS _(1460 μL)_ + L1 _(40 μL)_		✓
3	DS _(1000 μL)_ + L2 _(500 μL)_		✓
4	DS _(1420 μL)_ + *M. bovis*_(40 μL)_ + L1 _(40 μL)_	✓	✓
5	DS _(960 μL)_ + *M. bovis*_(40 μL)_ + L2 _(500 μL)_	✓	✓
6	SP4 _(1420 μL)_ + *M. bovis*_(40 μL)_ + L1 _(40 μL)_	✓	✓
7	SP4 _(960 μL)_ + *M. bovis*_(40 μL)_ + L2 _(500 μL)_	✓	✓
8	DS _(1436 μL)_ + *M. bovis*_(40 μL)_ + Enro _(24 μL)_	✓	
9	DS _(1436 μL)_ + *M. bovis*_(40 μL)_ + Doxy _(24 μL)_	✓	
10	SP4 _(1436 μL)_ + *M. bovis*_(40 μL)_ + Enro _(24 μL)_	✓	
11	SP4 _(1436 μL)_ + *M. bovis*_(40 μL)_ + Doxy _(24 μL)_	✓	

DS: Diluted semen. SP4: Specific medium for *Mycoplasma* spp. isolation (conditions without DS). L1: *Lactobacillus* spp. less concentrated (3.24 × 10^6^CFU/mL). L2: *Lactobacillus* spp. more concentrated (3.24 × 10^8^ CFU/mL). LAB: Lactic acid bacteria. ✓: Counts of *M. bovis* and lactic acid bacteria were made. *: Lactic acid bacteria were isolated in the pool of diluted semen. Enro: Enrofloxacin. Doxy: Doxycycline.

**Table 2:** Least squares means of the *Mycoplasma bovis* log colony-forming units (CFU)/mL, according to the studied analytical conditions. Conditions not contaminated with mycoplasmas are not included.

Condition	Description	Log CFU/mL of *M. bovis* ^1^
1	DS + *M. bovis*	6.71 ^a^
4	DS + *M. bovis*+ L1	4.55 ^b^
5	DS + *M. bovis +* L2	3.93 ^c^
6	SP4 + *M. bovis* + L1	7.73 ^d^
7	SP4 + *M. bovis +* L2	7.7 ^d^
8	DS + *M. bovis +* Enro	4.09 ^b,c^
9	DS + *M. bovis +* Doxy	3.72 ^c^
10	SP4+ *M. bovis +* Enro	3.43 ^c^
11	SP4+ *M. bovis +* Doxy	3.34 ^c^

Comparison of the means of *M. bovis* in all conditions, regardless of time. ^a, b, c, d^: Means with different superscripts between conditions differ significantly (*p <* 0.05). ^1^ Standard error of the mean (SEM): 0.23. DS: Diluted semen. SP4: Specific medium for *Mycoplasma* spp. isolation (conditions without DS). L1: *Lactobacillus* spp. less concentrated (3.24 × 10^6^ CFU/mL). L2: *Lactobacillus* spp. more concentrated (3.24 × 10^8^ CFU/mL). Enro: Enrofloxacin. Doxy: Doxycycline.

**Table 3:** Least squares means of the pH and log colony-forming units (CFU)/mL of *Mycoplasma bovis* and lactic acid bacteria, according to the analytical conditions studied by time.

Condition	Description	Hour (h)	pH ^1^	Log CFU/mL of *M. bovis* ^2^	Log CFU/mL of LAB ^3^
1	DS + *M. bovis*	0	6.93 ^a^	7.53 ^a^	0 ^a^
		15	6.74 ^a^	5.89 ^b^	3.82 ^a^
2	DS + L1	0	6.93 ^a^	---	3.82 ^a^
		15	6.73 ^a^	---	4.03 ^a^
3	DS + L2	0	6.93 ^a^	---	3.20 ^a^
		15	6.75 ^a^	---	3.91 ^a^
4	DS + *M. bovis* + L1	0	6.93 ^a^	7.55 ^a^	2.73 ^a^
		15	6.74 ^a^	1.55 ^b^	1.97 ^a^
5	DS + *M. bovis +* L2	0	6.93 ^a^	7.08 ^a^	4.23 ^a^
		15	6.50 ^b^	0.78 ^b^	13.62 ^b^
6	SP4 *+ M. bovis* + L1	0	7.68 ^a^	7.50 ^a^	4.38 ^a^
		15	7.32 ^b^	7.95 ^a^	2.06 ^a^
7	SP4 + *M. bovis +* L2	0	7.68 ^a^	7.22 ^a^	3.01 ^a^
		15	7.21 ^b^	8.19 ^b^	3.23 ^a^
8	DS + *M. bovis +* Enro	0	6.93 ^a^	7.59 ^a^	---
		15	6.74 ^a^	0.60 ^b^	---
9	DS + *M. bovis +* Doxy	0	6.93 ^a^	7.45 ^a^	---
		15	6.74 ^a^	0 ^b^	---
10	SP4+ *M. bovis +* Enro	0	7.68 ^a^	7.11 ^a^	---
		15	7.58 ^a^	0 ^b^	---
11	SP4+ *M. bovis +* Doxy	0	7.68 ^a^	6.93 ^a^	---
		15	7.68 ^a^	0^b^	---

^a, b^: Means with different superscripts between the times in all conditions differ significantly (*p* < 0.05). ^1^ Standard error of the mean (SEM): 0.08; ^2^ SEM: 0.31; ^3^ SEM: 1.63. DS: Diluted semen. SP4: Specific medium for *Mycoplasma* spp. isolation (conditions without DS). L1: *Lactobacillus* spp. less concentrated (3.24 × 10^6^ CFU/mL). L2: *Lactobacillus* spp. more concentrated (3.24 × 10^8^ CFU/mL). LAB: Lactic acid bacteria. Enro: Enrofloxacin. Doxy: Doxycycline. The pH values of the pools of DS and SP4 were considered as the initial pH (h0) of the conditions prepared in DS and SP4, respectively. The pH was measured in every condition separately at h15.
